# Change of cervical flexion range of motion influences postoperative sagittal alignment of the cervical spine after laminoplasty

**DOI:** 10.1186/s12893-024-02431-1

**Published:** 2024-05-14

**Authors:** Chengxin Liu, Xiangyu Li, Wei Wang, Bin Shi, Shibao Lu

**Affiliations:** 1 Department of Orthopedics, Xuanwu Hospital, Capital Medical University, Beijing, China; 2 National Clinical Research Center for Geriatric Diseases, Beijing, China

**Keywords:** Cervical laminoplasty, Cervical sagittal alignment, Cervical spondylotic myelopathy, Cervical spine range of motion, Change of cervical flexion range of motion

## Abstract

**Objective:**

The relationships between preoperative cervical spine range of motion (ROM) and postoperative cervical sagittal alignment (CSA), and clinical outcomes after laminoplasty (LMP) have been widely studied. However, the impact of ROM changes on postoperative CSA and clinical outcomes after LMP remains unclear. Herein, patients with cervical spondylotic myelopathy (CSM) were retrospectively analyzed to explore the association between postoperative cervical ROM changes and CSA and surgical outcomes.

**Methods:**

Patients who underwent cervical LMP at our hospital between January 2019 to June 2022 were retrospectively reviewed. CSA parameters were measured before the surgery and at the final follow-up. Loss of cervical lordosis (LCL) was defined as preoperative cervical lordosis (CL) - postoperative CL. An increase in the cervical sagittal vertical axis (I-cSVA) was defined as postoperative cervical sagittal vertical axis (cSVA) - preoperative cSVA. We defined the changes in cervical flexion range of motion (△Flex ROM, preoperative Flex ROM minus postoperative Flex ROM) > 10° as L- Flex ROM group, and △Flex ROM ≤ 10° as S- Flex ROM group. Japanese Orthopedic Association (JOA) score and visual analog score (VAS) were used to assess the surgical outcomes.

**Results:**

The study comprised 74 patients and the average follow-up period was 31.83 months. CL, total ROM, and Flex ROM decreased and cSVA increased after cervical LMP. LCL and I-cSVA were positively correlated with △Flex. Multiple linear regression analysis showed that a decrease in the Flex ROM was a risk factor for LCL and I-cSVA after LMP. LCL and I-cSVA were higher in the L-Flex ROM group than in the S-Flex ROM group. Postoperative JOA and the JOA recovery rate were worse in the L-Flex ROM group than in the S-Flex ROM group.

**Conclusions:**

Cervical total and Flex ROM decreased after cervical LMP. The reduction of Flex ROM was associated with LCL and I-cSVA after surgery. The preservation of cervical Flex ROM helps maintain CSA after LMP. Therefore, more attention should be paid to maintaining cervical ROM to obtain good CSA and surgical effects after cervical LMP.

## Introduction

Cervical laminoplasty (LMP) is a developed posterior approach surgical procedure for the treatment of cervical spondylotic myelopathy (CSM), with excellent surgical outcomes [1, 2]. The decompression effects provided by LMP mainly include direct posterior decompression through lamina lifting and indirect anterior decompression through cord posterior shifting. Therefore, it is very important to maintain adequate sagittal alignment of the cervical spine to obtain sufficient space for the shifting of the spinal cord.

The cervical spine range of motion (ROM) refers to the change in cervical lordosis (CL) in flexion and extension compared with the neutral position. Recent studies have reported that preoperative cervical ROMs are important predictors for changes in cervical sagittal alignment (CSA) and clinical outcomes after cervical LMP [3–9]. Fujishiro et al. [4] speculated that a large cervical flexion range of motion (Flex ROM) indicated that posterior neck muscular-ligament complex (PMLC) forces restricting motion toward the kyphotic position were weak, and Lee et al [5] thought that a small cervical extension range of motion (Ext ROM) indicated the low contraction reserve of PMLC. Although cervical LMP is a non-fusion procedure, cervical ROM also reduces after surgery [10–16]. As far as we know, some studies have reported the factors associated with the loss of motion after cervical LMP [10, 14, 17]. However, the impact of ROM changes on postoperative CSA and clinical outcomes after LMP remains elusive.

Herein, patients with CSM were retrospectively analyzed to explore the changes in cervical Flex and Ext ROM after cervical LMP. Meanwhile, this study explored the relationship between changes in postoperative cervical ROM and postoperative CSA and surgical outcomes. It was hypothesized that postoperative cervical ROM reduces, and excessive loss of cervical ROM is associated with cervical sagittal malalignment as well as poor clinical outcomes.

## Materials and methods

### Patient enrollment

Following the receipt of IRB approval (IRB number: 2018-086). Patients who underwent cervical LMP for CSM at our hospital between January 2019 to June 2022 were retrospectively analyzed. The exclusion criteria were as follows: a history of cervical spine surgery or fracture, significant spondylolisthesis, continuous ossification of the posterior longitudinal ligament, decompression levels including C2 or thoracic spine levels, less than 3 levels of cervical LMP or twelve months of follow-up, without complete radiographic or clinical data, and with postoperative complications during follow-up, such as stroke, lower extremity arterial disease.

### Surgical procedures

All patients were placed in a prone position on the operating table after general anesthesia with endotracheal intubation. An incision was made at the back of the neck and the paravertebral muscles were carefully separated from the spinous process and lamina while protecting the joint capsule. All patients underwent open-door posterior decompression using a mini titanium plate system. One side of the lamina was opened, while the other side acted as a hinge [18]. All patients were required to wear collars for 3–4 weeks after surgery. All patients did not receive systematic rehabilitation exercise after surgery.

### Radiological and clinical parameters

**Fig. 1:**
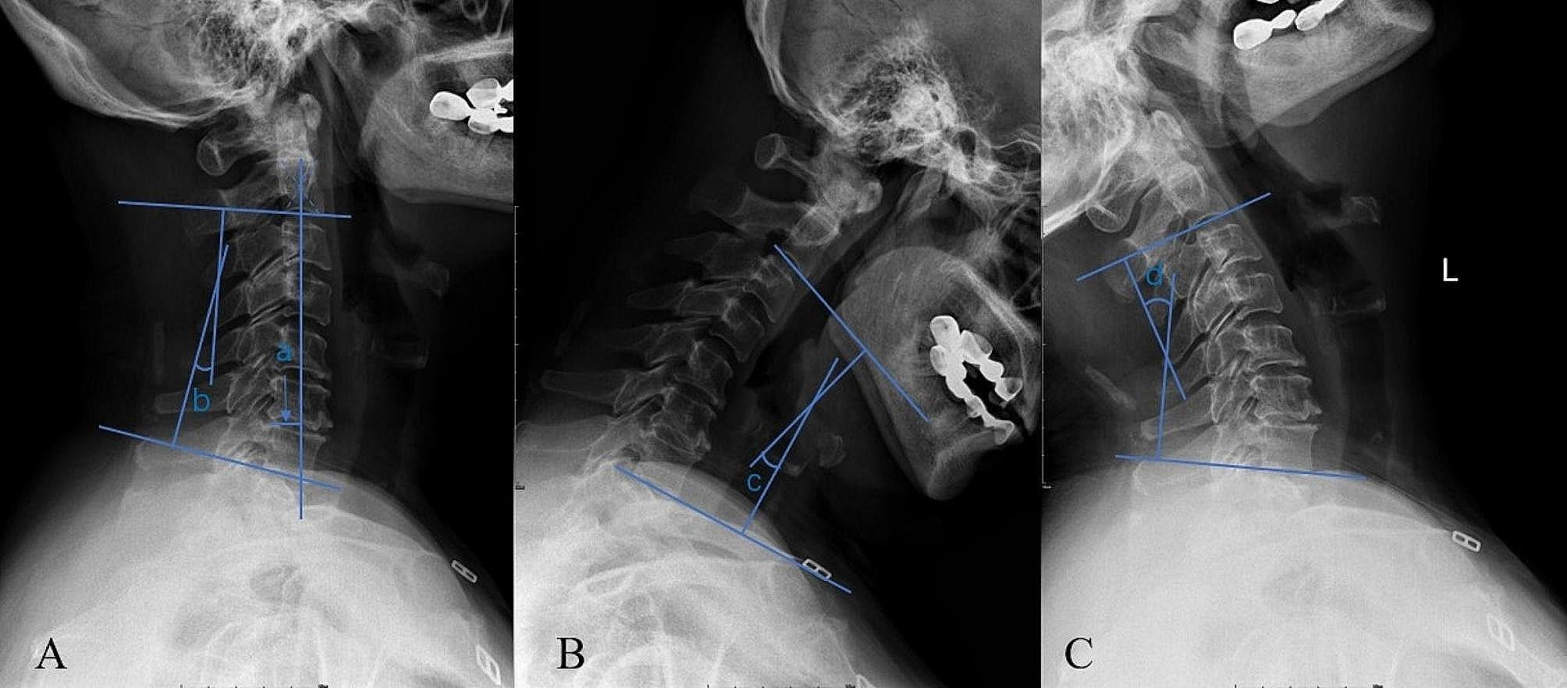
**A**, cervical X-ray in the neutral position: cSVA **(a)**, the horizontal distance from plumbline dropped from the C2 vertebral body to the posterosuperior corner of the C7 vertebra; CL **(b)**, the angle between the C2 lower endplate and the C7 lower endplate; **B** and **C**, cervical X-ray in the flexion and extension position: Flex CL **(c)** and Ext CL **(d)** refer to CL in flexion and extension positions. cSVA, cervical sagittal vertical axis; CL, cervical lordosis; Flex CL, cervical lordosis in flexion; Ext CL, cervical lordosis in extension

**Fig. 2:**
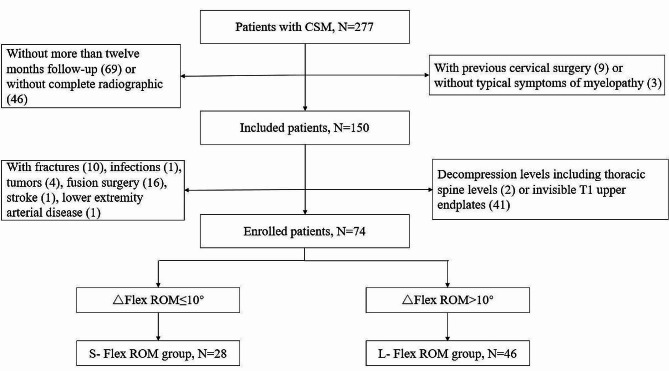
The flowchart of study. CSM: cervical spondylotic myelopathy, △Flex ROM: changes of cervical flexion range of motion

Radiological data were obtained on preoperative and follow-up X-rays, including CL, CL in flexion (Flex CL), CL in extension (Ext CL), and cervical sagittal vertical axis (cSVA) (Fig. 1). The cervical ROM was defined as Ext CL - Flex CL. The cervical Flex ROM was defined as CL - Flex CL. The cervical Ext ROM was defined as Ext CL - CL. △ indicated the changes (preoperation minus postoperation). Loss of cervical lordosis (LCL) was defined as preoperative CL - postoperative CL. An increase in cSVA (I-cSVA) was defined as postoperative cSVA - preoperative cSVA. All radiological parameters were performed two times for each patient by two surgeons separately and the average value was used for statistical analyses. Patients were divided into groups L- Flex ROM (△Flex ROM > 10°) and S- Flex ROM (△Flex ROM ≤ 10°) according to the changes in postoperative Flex ROM. The Japanese Orthopedic Association (JOA) score and visual analog score (VAS) were used to assess the surgical outcomes. JOA recovery rate was calculated as follows: 100 × (postoperative JOA - preoperative JOA)/ (17 - preoperative JOA). The flowchart of the study is depicted in Fig. 2.

### Statistical analysis

All data were analyzed using SPSS version 22.0 software (SPSS, Inc., Chicago, IL, USA). The variables were expressed as mean ± standard deviation. Pearson correlation analysis was used to examine the correlations between the variations in CSA and cervical ROM. Multiple linear regression models were conducted to identify the independent association between changes in cervical ROM and LCL and I-cSVA. T-tests and Mann-Whitney tests were performed to compare radiographic and clinical parameters between two groups. Statistical significance was defined as a p-value of less than 0.05.

## Results

### Patient characteristics

A total of 74 patients were recruited (mean age 61.49 ± 9.22 years; 52 males and 22 females). The average follow-up time was 31.83 (range 12–49) months. Patient demographics, surgery segments, and surgical outcomes are shown in Table 1. The reasons for the loss of follow up were as follows: died: 1, geographical reasons: 44, loss of contact: 15, old patients living alone: 9.

**Table 1:** Baseline characteristics of patients (*n* = 74)

Demographic		
**Age (yrs)**		61.49±9.22
**Sex**	Male	52 (70.27%)
	Female	22 (29.73%)
**BMI (kg/m** ^**2**^ **)**		26.62 ± 3.38
**Surgery segment (n)**		3.89 ± 0.81
**Follow up time (months)**		31.83±10.95
**JOA**	Pre	13.12±1.29
	Post	15.51±1.42
	recovery rate (%)	63.48±34.99
**VAS (neck)**	Pre	2.41±2.09
	Post	1.33±1.29

BMI: body mass index, JOA: Japanese Orthopedic Association, VAS: visual analogue score

### Comparison of pre- and post-operative dynamic and static parameters of the cervical spine

CL decreased and cSVA increased after cervical LMP (Table 2). Total ROM and Flex ROM decreased postoperatively, while Ext ROM remained unchanged after surgery (postoperative Ext ROM decreased in 51.35% (38/74) of the patients but increased 48.65% (36/74) of the patients).

**Table 2:** Comparison of sagittal parameters between pre-operation and post-operation (*N* = 74)

Parameters	Pre-operation	Post-operation	*P* value
**CL**	12.91±8.67	6.84±11.50	0.000
**cSVA**	20.93±8.31	27.41±11.20	0.000
**Total ROM**	38.31±9.21	26.74±9.86	0.000
**Flex ROM**	29.50±8.04	17.44±8.91	0.000
**Ext ROM**	8.81±5.63	9.30±5.83	0.685

CL: cervical lordosis, cSVA: cervical sagittal vertical axis, total ROM: cervical spine range of motion, Flex ROM: cervical spine range of flexion, Ext ROM: cervical spine range of extension

### Correlation analysis

Correlation analysis showed that LCL was negatively correlated with △Ext ROM (*r* = -0.588, *p* < 0.01) and positively correlated with △Flex (*r* = 0.653, *p* < 0.01). No significant correlation was observed between LCL and △total ROM. I-cSVA was positively correlated with △total ROM (*r* = 331, *p* < 0.05) and △Flex (*r* = 373, *p* < 0.05). No significant correlation was observed between I-cSVA and △Ext ROM (Table 3).

**Table 3:** Correlation analysis (*N* = 74)

Parameters	LCL	I-cSVA
**ΔTotal ROM**	0.265	0.331^*^
**ΔExt ROM**	-0.588^@^	-0.061
**ΔFlex ROM**	0.653^@^	0.373^*^

*p < 0.05, ^@^*p* < 0 0.01 statistically significant difference

LCL: loss of cervical lordosis, I-cSVA: increase in cervical sagittal vertical axis, △ indicates the changes (preoperation minus postoperation), total ROM: cervical spine range of motion, Flex ROM: cervical spine range of flexion, Ext ROM: cervical spine range of extension

### Risk factors for LCL and I-cSVA

Multiple linear regression analysis was performed using variables that demonstrated a significant correlation with the LCL or I-cSVA (Table 4). The results showed that LCL decreased by 0.413° (*p* = 0.001) for each change in Ext ROM and increased by 0.333° (*p* = 0.000) for each change in Flex ROM. LCL was predicted using the following regression equation: LCL = 0.333 △Flex − 0.413 △Ext ROM. I-cSVA increased by 0.305 mm for each change in Flex ROM (*p* = 0.025). I-cSVA was predicted using the following regression equation: I-cSVA = 0.305△Flex. No independent association was found between I-cSVA and △Ext ROM (*p* = 0.707).

**Table 4:** Multiple linear regression model shows correlations between LCL, I-cSVA and the changes in cervical motion (*n* = 74)

Model	Unstandardized coefficients		Standardized coefficient	T	Sig	*R* ^2^
	B	SE	β			
**LCL**						0.588
(Constant)	1.850	1.171		1.580	0.123	
△Ext ROM (°)	-0.413	0.113	-0.423	-3.648	**0.001**	
△Flex ROM (°)	0.333	0.074	0.519	4.474	**0.000**	
**I-cSVA**						0.143
(Constant)	-0.160	2.044		-0.078	0.938	
△Ext ROM (°)	0.075	0.198	0.063	0.379	0.707	
△Flex ROM (°)	0.305	0.130	0.393	2.350	**0.025**	

LCL: loss of cervical lordosis, I-cSVA: increase in the cervical sagittal vertical axis, △ indicates the changes (preoperation minus postoperation), Flex ROM: cervical spine range of flexion, Ext ROM: cervical spine range of extension

### Comparison of evaluated parameters based on △Flex ROM

Compared with the S-Flex ROM group, postoperative LCL was significantly higher and postoperative JOA and the JOA recovery rate were significantly lower in the L-Flex ROM group. I-cSVA was higher in the L-Flex ROM group compared with the S-Flex ROM group but with no significant statistical significance (9.28 ± 9.11 VS 1.89 ± 6.60, *p* = 0.089) (Table 5).

**Table 5:** Comparison of evaluated parameters according to the changes of postoperative Flex ROM

Parameters	L- Flex ROM (*n* = 46)	S- Flex ROM (*n* = 28)	*P* value
**LCL**	8.57±4.69	1.94±8.52	**0.007**
**I-cSVA**	9.28±9.11	1.89±6.60	0.089
**Pre-JOA**	13.00±1.41	13.31±1.11	0.876
**Post-JOA**	15.03±1.53	16.31±1.26	**0.005**
**JOA recovery rate**	51.33±32.55	82.21±29.92	**0.008**
**Pre-VAS (neck)**	2.46±2.28	2.33±2.23	0.984
**Post-VAS (neck)**	1.52±1.31	1.06±1.05	0.288

LCL: loss of cervical lordosis, I-cSVA: increase in the cervical sagittal vertical axis, JOA: Japanese Orthopedic Association, VAS: visual analog score

### Representative cases

**Fig. 3:**
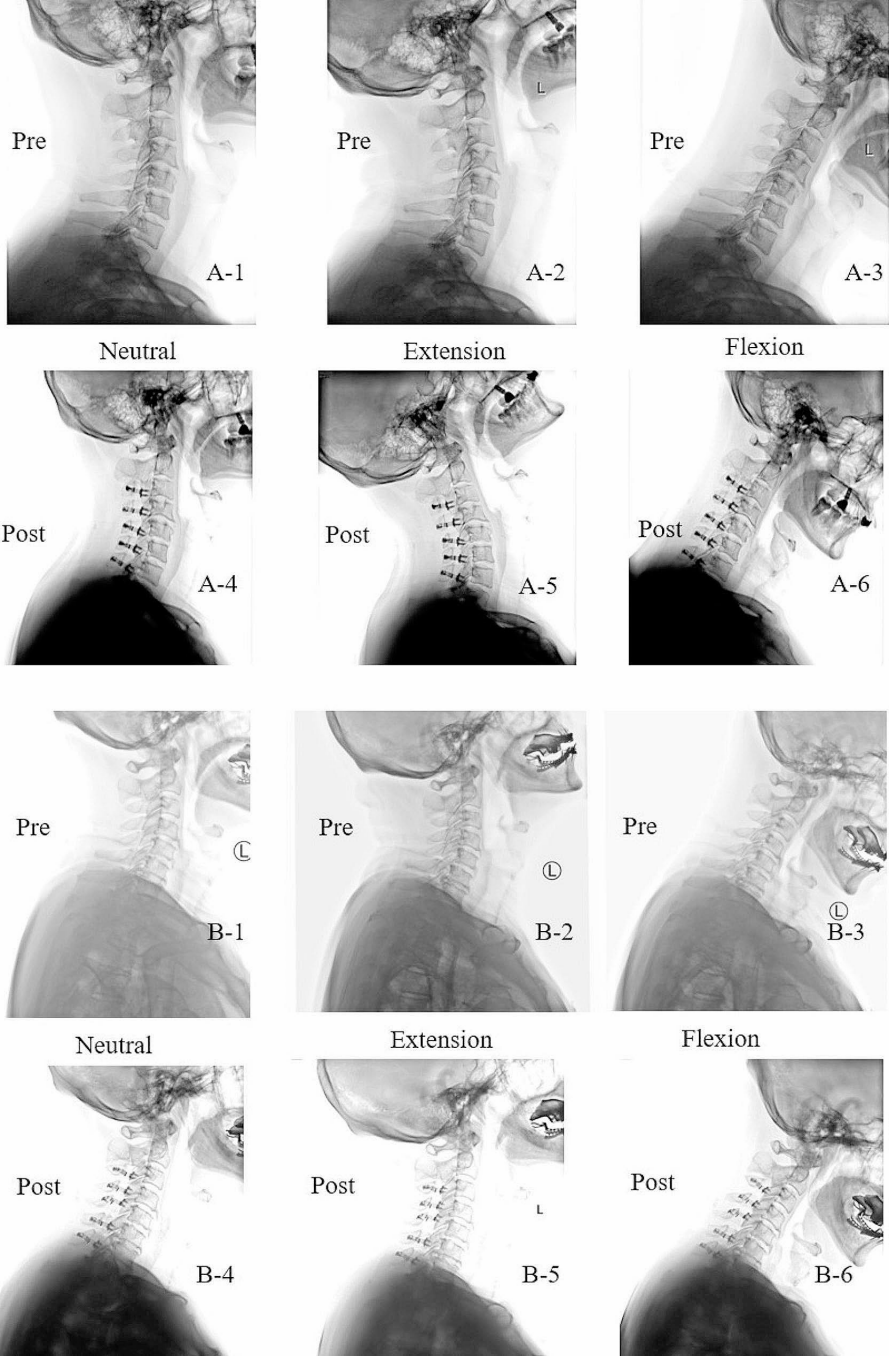
Representative images of case

Two representative cases are shown in Fig. 3. Figure 3-A. shows a patient in the S-Flex ROM group. A 46-year-old man who underwent laminoplasty of C3-7. After 34 months of surgery, no significant changes were observed in CL, cSVA, and Flex ROM. Pre-JOA was 13, post-JOA was 17, and the JOA recovery rate was 100%. No neck pain was reported before and after surgery. Figure 3-B shows a patient in the L-Flex ROM group. A 66-year-old man who underwent laminoplasty of C3-7. After 23 months of surgery, Flex ROM and CL decreased significantly, and cSVA increased significantly. Pre-JOA was 12, post-JOA was 14, and the JOA recovery rate was 40%. A mild axial symptom (pain around the posterior neck and suprascapular areas) occurred after surgery.

## Discussion

A reduction in cervical ROM is one of the common complications after LMP [13, 14, 19]. C3 laminoplasty, disruption of subaxial deep extensors, postoperative intervertebral fusion, and prolonged cervical collar use are potential risk factors for loss of ROM [10, 12, 20, 21]. The current study found that total cervical ROM decreased at a mean of 32 months follow-up after LMP, which was mainly due to the decrease in Flex ROM. Cervical Flex ROM decreased in most patients (89.19%); however, only half of the patients (51.35%) had a reduction in Ext ROM after LMP. Most studies have reported the loss of total cervical ROM after LMP but the change in Ext ROM remains controversial. Some studies found a significant decrease in Ext ROM after cervical LMP [13], while others showed that Ext ROM remained stable [21] or even increased segmentally after surgery [22]. We speculate that LMP can lead to contracture and bony union of posterior cervical structures, which might restrict postoperative Flex ROM rather than Ext ROM.

Most clinical studies investigating the association between cervical ROM and CSA focused on the prognostic effects of preoperative cervical ROM on postoperative LCL. Lee et al [5] and Ren et al [7] found that cervical Ext ROM was a predictor of kyphotic alignment change after LMP and significant LCL occurred much more frequently in patients whose Ext ROM was < 14° and cervical kyphosis occurred much more frequently in patients whose Ext ROM was < 22.1°. Meanwhile, a greater gap or ratio between Flex ROM and Ext ROM was also associated with severe LCL [3, 6, 23]. The present study found that postoperative CL decreased and cSVA increased after cervical LMP, which is consistent with previous studies [24–27]. Meanwhile, a significant correlation between the decrease in Flex ROM and cervical sagittal malalignment after LMP was also registered. The multiple linear regression model indicated that a reduction in Flex ROM was the risk factor for LCL and I-cSVA. It was found that the more Flex ROM decreased, the more postoperative LCL and I-cSVA occurred. As a posterior approach, LMP can damage the muscles, ligaments, and bones of the cervical spine. This procedure can cause postoperative contracture of posterior cervical structures and bony fusion, which are closely associated with the reduction in Flex ROM [17, 22]. Additionally, excessive reduction in Flex ROM aggravates LCL and I-cSVA after surgery.

As a common surgical procedure for the treatment of CSM, the surgical outcomes of LMP are not always satisfactory for all patients. Insufficient postoperative CL due to severe LCL and postoperative cervical sagittal imbalance due to severe I-cSVA are associated with poor recovery of neurological function and neck pain [3, 24–26, 28–31]. Our data showed that compared with △Ext ROM, △Flex ROM had a larger influence on cervical sagittal malalignment after surgery. Therefore, we divided patients into two groups based on the changes in Flex ROM after cervical LMP: L-Flex ROM (△Flex ROM > 10°) and S-Flex ROM (△Flex ROM ≤ 10°). The results showed that the neurological function and neck pain recovered in the two groups after surgery, but with varying degrees of recovery. Compared with the S-Flex ROM group, the L-Flex ROM group exhibited a worse post-JOA and JOA recovery rate. Furthermore, patients in the S-Flex ROM group had better relief of neck pain but with no statistical significance (S-Flex ROM group: deterioration of neck pain occurred in 3.57% (1/28) of the patients, L-Flex ROM group: deterioration of neck pain occurred in 13.04% (6/46) of the patients, *p* = 0.242). These results were analogous to those obtained by Chen et al. [21], who reported that better clinical outcomes occurred in patients with stable CL, cSVA, and Flex ROM after cervical LMP. These clinical outcomes may be explained by several factors. Firstly, the degeneration of the posterior cervical structure is more serious in patients with excessive loss of cervical Flex ROM after LMP and the contraction reserve is less. Secondly, insufficient CL and cervical sagittal imbalance are the reasons for insufficient posterior drift space of the spinal cord after surgery. Finally, an increase in cSVA may lead to an increase in posterior cervical stress, which maintains the cervical sagittal balance.

According to these findings, we speculate that the preservation of postoperative CSA is not primarily due to intervertebral soft tissue contracture or bony fusion. Instead, it appears to be maintained by dynamic factors such as muscles or ligaments. We believe that the less decrease in cervical Flex ROM and the better preservation of cervical flexibility are associated with the maintenance of CSA after LMP. Therefore, early removal of cervical collars and early postoperative systematic rehabilitation exercise to restore the flexibility of the cervical spine is significantly important. Stable dynamic and static sagittal alignment of the cervical spine help obtain good surgical effects after LMP.

Nonetheless, this study has several limitations. First, since this is a retrospective study, a selection bias may exist. Meanwhile, the patients in this study are mainly elderly patients from different parts of the country, so some patients lost to follow up. In fact, we made great efforts to promote the level of the quality of follow-up, including pre-discharge education and multiple out-of-hospital contacts. Second, this study did not evaluate the cervical paraspinal muscles. We believe that cervical ROMs are also valuable in estimating the cervical functional reserve, corresponding to measuring muscle volume. Thus, the results of this study have a certain reference significance.

## Conclusions

Cervical total and Flex ROM decreased after cervical LMP. The reduction in Flex ROM was associated with LCL and I-cSVA after surgery. The preservation of cervical Flex ROM helps maintain CSA after LMP. Therefore, more attention should be paid to maintaining cervical ROM to obtain good CSA and surgical effects after cervical LMP.

## Data Availability

All data generated or analyzed during this study are included in this published article.

## Abbreviations

LMP: Laminoplasty
CSM: Cervical spondylotic myelopathy
CL: Cervical lordosis
cSVA: Cervical sagittal vertical axis
Flex CL: CL in flexion
Ext CL: CL in extension
ROM: Cervical spine range of motion
Flex ROM: Cervical flexion range of motion
Ext ROM: Cervical extension range of motion
CSA: Cervical sagittal alignment
LCL: Loss of cervical lordosis
I-cSVA: Increase in the cervical sagittal vertical axis
JOA: Japanese Orthopedic Association score
VAS: Visual analog score
PMLC: Posterior neck muscular-ligament complex
BMI: Body mass index
